# A new strategy for faster urinary biomarkers identification by Nano-LC-MALDI-TOF/TOF mass spectrometry

**DOI:** 10.1186/1471-2164-9-541

**Published:** 2008-11-14

**Authors:** K Benkali, P Marquet, JP Rérolle, Y Le Meur, LN Gastinel

**Affiliations:** 1 INSERM U850, France; Univ. of Limoges, France; CHU Limoges, France; 2 Department of Nephrologie-Transplantation, University Hospital, Limoges, France; 3 Department of Nephrologie-Transplantation, University Hospital, Brest, France

## Abstract

**Background:**

LC-MALDI-TOF/TOF analysis is a potent tool in biomarkers discovery characterized by its high sensitivity and high throughput capacity. However, methods based on MALDI-TOF/TOF for biomarkers discovery still need optimization, in particular to reduce analysis time and to evaluate their reproducibility for peak intensities measurement. The aims of this methodological study were: (i) to optimize and critically evaluate each step of urine biomarker discovery method based on Nano-LC coupled off-line to MALDI-TOF/TOF, taking full advantage of the dual decoupling between Nano-LC, MS and MS/MS to reduce the overall analysis time; (ii) to evaluate the quantitative performance and reproducibility of nano-LC-MALDI analysis in biomarker discovery; and (iii) to evaluate the robustness of biomarkers selection.

**Results:**

A pool of urine sample spiked at increasing concentrations with a mixture of standard peptides was used as a specimen for biological samples with or without biomarkers. Extraction and nano-LC-MS variabilities were estimated by analyzing in triplicates and hexaplicates, respectively. The stability of chromatographic fractions immobilised with MALDI matrix on MALDI plates was evaluated by successive MS acquisitions after different storage times at different temperatures.

Low coefficient of variation (CV%: 10–22%) and high correlation (R^2 ^> 0.96) values were obtained for the quantification of the spiked peptides, allowing quantification of these peptides in the low fentomole range, correct group discrimination and selection of "specific" markers using principal component analysis. Excellent peptide integrity and stable signal intensity were found when MALDI plates were stored for periods of up to 2 months at +4°C. This allowed storage of MALDI plates between LC separation and MS acquisition (first decoupling), and between MS and MSMS acquisitions while the selection of inter-group discriminative ions is done (second decoupling). Finally the recording of MSMS spectra to obtain structural information was focused only on discriminative ions in order to minimize analysis time.

**Conclusion:**

Contrary to other classical approaches with direct online coupling of chromatographic separation and on the flight MS and/or MSMS data acquisition for all detected analytes, our dual decoupling strategy allowed us to focus on the most discriminative analytes, giving us more time to acquire more replicates of the same urine samples thus increasing detection sensitivity and mass precision.

## Background

Non invasive monitoring of the kidney status using urine biomarkers can lead to the early diagnosis of renal dysfunction, which might help avoid or diminish the use of invasive methods like renal biopsy and improve patients treatment and survival particularly in renal or uro-genital pathologies such as renal cancers, diabetic nephropathy or allograft dysfunctions in renal transplantation.

Several urine biomarker discovery studies in renal diseases have been published, most of which used Surface Enhanced Laser Desorption Ionization-Time of Flight Mass spectrometry (SELDI-TOF MS) which is characterized by its poor resolution but high throughput capacity [1-3]. Recently, Capillary Electrophoresis-Mass Spectrometry (CE-MS) has been largely used in urinary peptidomic studies for biomarkers discovery, giving rise to many published results though sometimes without protein or peptide identification [4-6]. Also, different candidate biomarkers have thus been proposed for the same pathology, none of which have been validated in a large population. Such diversity may be due to the inter- and intra-individual variability in the urine proteome, and/or to differences in urine sampling, storage and analysis procedures across different studies [7]. Some authors gave recommendations for the standardization of proteomic analysis procedures, particularly for the pre-analytical phase (sample collection, storage, and preparation). Preventive measures must be taken to avoid alterations of the urinary proteome and peptidome composition due to bacterial growth [8], storage conditions, freeze-thaw cycles, pH conditions, etc [8-11].

LC-MALDI-TOF/TOF analysis is a new potential tool for biomarkers discovery because of its high sensitivity and high throughput capacity [12]. However, the use of MALDI-TOF/TOF as an analytical tool in clinical research still needs optimization and evaluation [13]. One of the major limitations of the MALDI-based technique is its relative poor reproducibility in measuring m/z abundances (peak intensity), which may be essential in biomarker discovery where all-or-nothing variations are probably not the most frequent [14]. In addition, LC-MALDI-TOF/TOF analysis generally takes a long time, which is hardly practical when analysing large numbers of samples. However, this technique is recognized for its high mass precision (typically in the range of 5 ppm to 20 ppm routinely).

The aims of the present study were: (i) to optimize and critically evaluate each step of a comprehensive urine biomarker discovery method based on Nano-LC coupled off-line to MALDI-TOF/TOF, taking full advantage of the dual decoupling between Nano-LC, MS and MS/MS to reduce the overall analysis time and increase the technique throughput, as will be requested during the validation phase involving a larger population set; (ii) to evaluate the quantitative performance and reproducibility of nano-LC-MALDI analysis in biomarker discovery; and (iii) to evaluate the robustness of biomarkers selection using pseudo-biomarker peptides.

## Methods

### Sample collection

Urine samples from 3 healthy volunteers who had not recently received any medication were used. All subjects gave their written informed consent to participate in this study. A sample of 40 ml of mid-stream urine was collected in a polypropylene tube containing one COMPLETE™ Mini tablet (Roche Diagnostics, Mannheim, Germany) to inactivate endogenous and exogenous proteases, and sodium azide at a final concentration of 2 mg/ml to prevent bacterial growth. Cells or other insoluble materials (debris, crystals and aggregated materials) were cleared from the samples by centrifugation at low speed (2500 rpm, 1 h at 4°C), then 5 ml urine sample aliquots were transferred in polypropylene tubes, lyophilized, and stored at -80°C until their usage.

### Urine sample processing

Before nanoLC-MALDI MS analysis, each stored sample of lyophilized urine was reconstituted with 2 ml of 0.1% trifluoro-acetic acid (TFA) (Sigma-Aldrich, St Louis, MO, USA) in HPLC grade water and submitted to solid phase extraction (SPE) on a C2 ethyl 2.5 mL cartridge (Amersham Biosciences, Buckinghamshire, England), following the standard procedure recommended by the vendor. Urinary peptides were eluted with acetonitrile/water (50/50, v/v) solution containing 0.5% formic acid. The eluate was finally evaporated using a SpeedVac^® ^system (Thermo-Fisher, Château Gontier, France) and then resuspended in 70 μL of 0.1% TFA. Peptides and proteins concentrations were measured using Bradford reagent (Sigma-Aldrich, St Louis, MO, USA). Then, 70 μl of the urine extract were mixed with 2 μl of the Proteomix 4 peptides calibrator (Laserbio, Sophia-Antipolis, France) consisting of: Human angiotensin II (1046.54 Da, at 21 pmol/μl); neurotensin (1672.91 Da, at 14 pmol/μl); ACTH 18–39 fragment (2465.19 Da, at 17.5 pmol/μl); and insulin β chain oxidized (3494.65 Da, at 87.5 pmol/μl). Peptide calibrators were used for subsequent time-shift alignment and intensity normalization of the Nano-LC-MALDI MS reconstituted chromatograms.

### Urinary peptides separation using Nano-HPLC

Chromatographic separation of peptides was performed using an Ultimate 3000 nano-HPLC system (LC Packings, DIONEX, Sunnyvale, USA) equipped with: a thermostated column compartment, a six-port micro-switching valve, a dynamic nanoflow splitter with a flow meter, a micro vacuum degasser and a thermostated microwell-plate autosampler with a six-port micro-switching valve. One to five microlitres of reconstituted urine containing the same relative quantity of materials (2 μg of peptides) were concentrated on a (0.5 cm × 300 μm i.d.) trapping column packed with C18 PepMap 100 (LC Packings DIONEX) using mobile phase A: acetonitrile/water (2:98, v/v) with 0.1% TFA delivered at 20 μl/min. The trapping column was switched on-line with the analytical column after 5 min loading time. Chromatographic separation of peptides was performed using a C18 PepMap 100 column (15 cm × 75 μm i.d., LC Packings DIONEX) and using a linear gradient of B, a mixture of acetonitrile/water (90:10) with 0.1% TFA, in solvent A, as follows: from 10% to 45% of B in 150 min, switched to 100% buffer B for 10 min, followed by 10 min re-equilibration with buffer A at a constant flow rate of 0.3 μl/min, stabilized by an active splitter (1:1000). The column was directly coupled to a UV flow cell detector (Ultimate DIONEX) at the exit of which the effluent was coaxially mixed with a solution of 3.5 mg/ml α-cyano-4-hydroxycinnamic acid MALDI matrix (α-CHCA) (Sigma-Aldrich, St Louis, MO, USA) prepared daily and delivered at a flow rate of 0.9 μL/min (3:1), then directed towards an on-line Probot (LC Packings DIONEX) plate-spotting system. Each spot represented a 12-second "fraction" (60 nL) of the reverse phase gradient. The triplicate runs of each sample, each containing 600 spots, were distributed on the same MALDI plate.

### MALDI-TOF/TOF data acquisition

The spots representing the different chromatographic fractions were analyzed using a 4800 MALDI-TOF/TOF mass spectrometer (Applied Biosystems/MDS Sciex, Toronto, Canada), equipped with a neodymium: yttrium-aluminum-garnet laser emitting at λ = 355 nm with a repetition rate of 200 Hz. The mass spectrometer was controlled by the 4000 Series Explorer, version 3.5.2 program. For MS analyses, typically 800 spectra were acquired for each spot in the reflector positive mode in the mass range of 800 to 5000 m/z, with 15 ppm mass tolerance (external calibration). The spots of fractions eluted before 40 min and after 160 min were not analyzed for the sake of time, as preliminary investigations showed that they apparently contained very little peptide information.

### Data processing and analysis

Peak lists obtained from Nano-LC-MALDI-TOF chromatograms were processed using MarkerView version 1.1 (Applied Biosystems/MDS Sciex, Toronto, Canada). Alignment was first performed with 45% retention time and 0.5 amu mass tolerances. These settings were optimized values found to give a maximum number of common peaks between sample replicates. Then, data were scaled in intensity by normalization to the median intensity of one reference chromatogram (chromatogram with the highest signal intensity) using MarkerView. After alignment and normalization, the data were filtered out from MALDI residual α-CHCA matrix clusters. As most matrix clusters had masses below 1000 m/z, filtration consisted in excluding all ions with m/z < 1000.

Principal component analysis (PCA) was performed on processed data using different scaling options (mean centre, autoscale, range scale, and Pareto scale [15]) proposed by MarkerView program. Pareto scaling was finally retained because of its best grouping performance explaining the largest variability in three principal components vectors (PC > 10%). Pareto data pre-treatment consists of mean centring and scaling by square root of standard deviation of m/z intensities. The ions were classified according to the sum of their loading values in the first three principal components PC1, PC2, and PC3. Ions with the highest PC loading values were considered as the most discriminative ions.

### MALDI-TOF/TOF MSMS data acquisition

**Figure 1:**
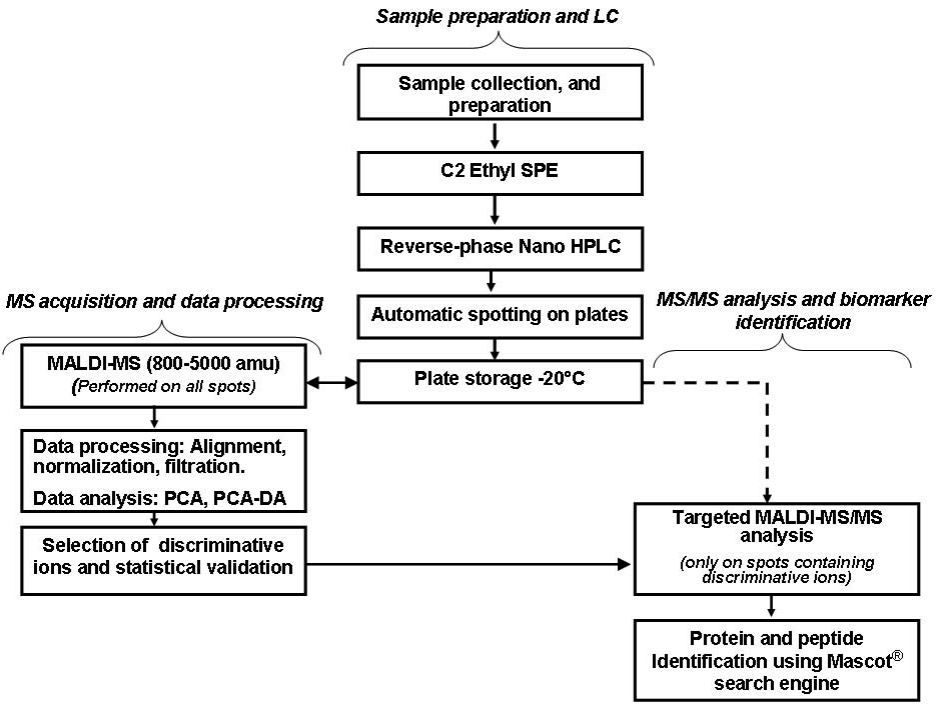
Workflow of the dual decoupling procedure based on Nano-LC-MALDI-TOF/TOF for the discovery of urine biomarkers.

MS/MS data acquisition was only performed on MALDI plate stored at -20°C, for peptide sequence and protein identification of these selected discriminative ions. The typical experiment workflow is summarized in Figure 1. MS/MS acquisitions were then carried out using air as collision gas at a pressure of ~3.0 × 10^-6 ^torr and collision energy of 1 kV. Approximately 2,000 spectra were added up for each spot. The peaks were de-isotoped and only those with s/n ≥ 10 were retained for interpretation.

### Protein identifications using MS and MSMS data

MSMS data were pre-processed by manufacturer computer program GPS Explorer version 3.6 (Applied Biosystems) and then identification of urine proteins from their peptides sequences was established using MASCOT search engine version 2.1 (Matrix Science, Boston, MA) with the last updated version of Swiss-Prot protein database merged with random sequences. Methionine oxidation and Asn and Gln deamidation were selected as the only variable modifications. The tolerance for precursor ion and MS/MS fragment mass values was set at 150 ppm and 0.5 Da, respectively. Only the 5 top-ranked peptide matches were taken into consideration for protein identification.

### Method validation

#### Extraction recovery

A pool of healthy volunteers' urine was divided in two aliquots which were spiked with the same amount of a mixture of four peptides (Human angiotensin II 1046.54 Da 60 pmol/μl; Neurotensin 1672.91 Da, 40 pmol/μl; ACTH 18–39 fragment 2465.19 Da, 50 pmol/μl; Insulin β chain oxidized 3494.65 Da, 250 pmol/μl), one aliquot before and the second after solid phase extraction (SPE) with the C2-ethyl cartridges. The samples were processed in triplicate to evaluate the reproducibility of sample processing and then analyzed in triplicate by nano-LC off-line MALDI TOF as described above.

#### Influence of storage conditions on peptides spotted on MALDI plates

The same mixture of four peptides was mixed with α-CHCA matrix solution at 3.5 mg/ml (1:1, v/v), and 1 μl was spotted manually 24 times on each of three MALDI plates. The plates were kept in the dark, one at room temperature, one at +4°C and the third at -20°C, for 2 months. Once a week over this 2 month period, 3 different spots per plate were analyzed using MALDI TOF/TOF in the reflector mode.

#### Quantitative response and performance of data processing and analysis

Quantitative accuracy and reproducibility of MALDI-TOF/TOF as well as the aptitude of the data pre-processing the data processing and the data analysis procedures to select discriminative ions were evaluated by comparing two samples prepared from C2-extract of a healthy volunteer's urine aliquot, spiked or not with increasing concentrations of three peptides: Human angiotensin II (1046.54 Da 1X = 120 fmol/μl), neurotensin (1672.91 Da, 1X = 80 fmol/μl) and ACTH 18–39 fragment (2465.19 Da, 1X = 100 fmol/μl). Six replicates of each spiked and non-spiked aliquots were then analyzed by LC-MALDI-TOF/TOF as described above, including retention time alignment and intensity normalization.

## Results and discussion

**Figure 2:**
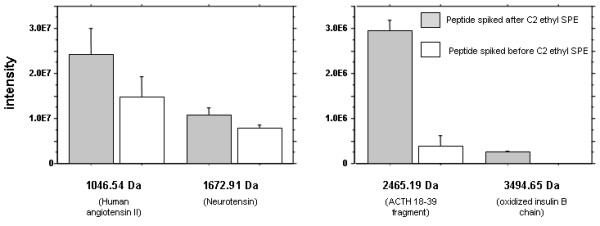
**Recovery of spiked peptides after C2-ethyl solid phase extraction of a healthy volunteer's urine sample.** Signal intensities of 4 standard peptides were evaluated before and after C2 ethyl SPE extraction. Error bars represented SD of triplicate measurements.

Solid phase extraction and desalting on C2-ethyl cartridges resulted in losses of large urinary peptides and proteins. The synthetic peptides were recovered with yields of approximately 60%, 80% and 20% for human angiotensin II (1046.54 Da), neurotensin peptide (1672.0 Da) and ACTH fragment (2465.19 Da), respectively, while the oxidized bovine insulin beta chain (3494.5 Da) was totally lost (Figure 2). Some peptides with m/z > 3000 Da could nevertheless be detected in urine samples processed this way (approximately 20% of the detected masses), meaning either that they were highly concentrated or that peptide loss is not only related to their size, but also to their structural properties. A good reproducibility of urine sample processing was obtained using the SPE C2 ethyl procedure for peptide extraction in spiked urine, showing coefficient of variation (CV %) of 14%, 20%, and 10% for the human angiotensin II (1046.54 Da), neurotensin peptide (1672.0 Da) and ACTH fragment (2465.19 Da), respectively. The non-hydrolyzed urinary peptidome was studied as a source of potential biomarkers instead of trypsinized urine proteins and peptides in order to reduce the complexity of analytes in urines, avoid protease contaminations and reveal potentially natural or pathologically induced protease activity in urine of patients following renal or uro-genital injury [1,16]. Sample desalting and peptide extraction was done on C2-ethyl cartridges that retain peptides based on their polarity and size characteristics. Moreover, this step removes most of intact proteins and consequently diminishes the large diluting effect caused by major serum proteins such as human serum albumin, possibly found in urine during physiological episodes of hypertension or other stresses/exercises, as well as in different renal diseases [7,17]. This sample preparation method was also chosen because of possible automation to increase the analytical throughput. The same resin was used extensively in formerly published studies describing urinary peptides as potential biomarkers [6,5].

A relatively long chromatographic run of 180 min was chosen in order to obtain the best separation of peptides without too much loss of sensitivity, resulting in a decrease of the number of urinary peptides immobilized per spot/fraction to be analyzed during MS data acquisition, Aligned and filtered urinary chromatograms contained 657 ions, the spiked peptides were eluted at 48.50 min, 43.17 min and 53.50 min retention time for m/z 1046.54, 1672.94, and 2465.19 respectively (additional file 1). As only the fractions between 40 and 160 min were spotted, it resulted in approximately 600 spots per replicate, so that full informative triplicate nanoLC chromatograms could be spotted on a single MALDI plate. This approach decreased plate storage space, cost and minimized plate-to-plate signal variability.

Targeted MSMS analysis (as described in figure 1) allows sequence peptides identification of spiked pseudo-biomarkers (additional files 2 to 7).

The MALDI-TOF/TOF MS analysis of one plate typically took 2.5 hours. While the unsupervised MS/MS analysis of an entire plate would have taken 16 hours, our actual targeted MS/MS analysis took 17 seconds per selected ion and per spot. Complete MS analysis of a urine sample in triplicate (3 × 600 spots) still took a long time but the decoupling between MS and MSMS resulted in a gain of time of approximately 10 fold.

**Figure 3:**
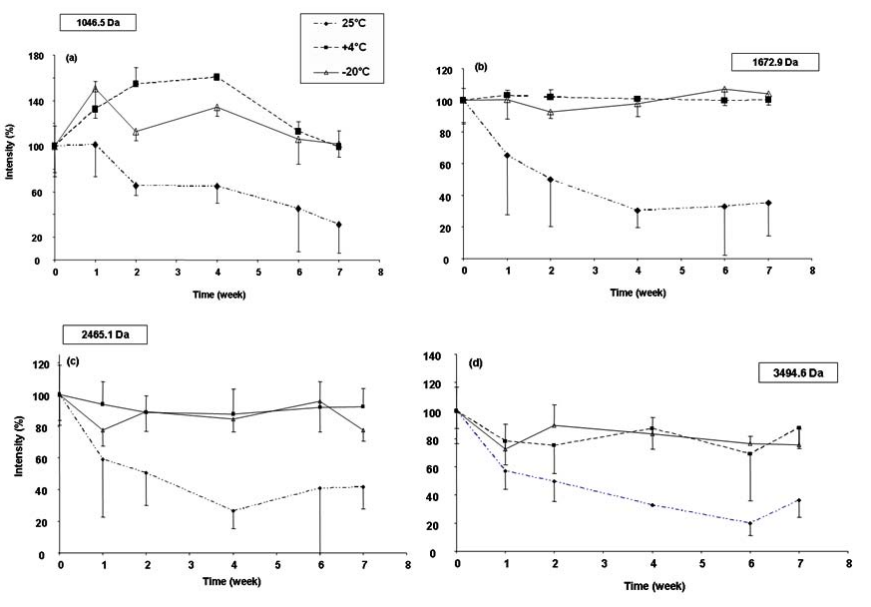
**Specific peptides MS signal intensity versus time after plate storage at 3 different temperatures (25°C, 4°C, -20°C)**. (a) human angiotensin II (MW = 1046.54), (b) neurotensin (MW = 1672.91), (c) ACTH fragment 18–39 (MW = 2465.19), and (d) oxidized insulin B chain (MW = 3494.65). Error bars represented SD from triplicate measurements.

Figure 3 shows that the MS intensity of spotted standard peptides is roughly stable over time when the plates were stored at +4°C or -20°C in the dark. At room temperature, MS intensity of the same peptides decreased by 60% over a few weeks for all peptides. The excellent structure integrity of peptides immobilized with the MALDI matrix on the plates stored in the dark at -20°C allowed us to take advantage of the decoupling between LC, MS, and MS/MS procedures. First, we performed MS data acquisition only on all 600 spots in triplicate for each sample, and secondly MS/MS data acquisition on the most discriminative ions selected after MS data analysis (20 to 50 ions). This approach resulted in a dramatic decrease in the MS/MS data acquisition time. However, the limiting factors of this process are: the cost of the MALDI plates; the amounts of immobilized analytes; and our ability to locate precisely selected analytes by their retention time on immobilized chromatogram fractions. Moreover, the immobilized urine samples were sufficiently stable on MALDI plates to consider sending the plates to different laboratories for cross-validation on different MALDI-TOF/TOF platforms.

**Figure 4:**
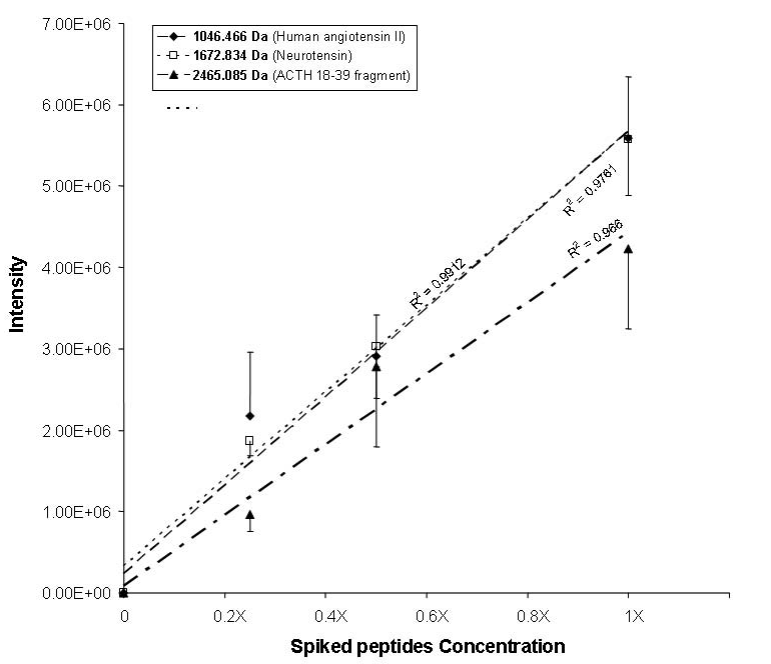
**Quantitative performance of MALDI-TOF/TOF for the determination of the following peptides spiked at increasing concentrations in blank urine: **Human angiotensin II (1046.54 Da, higest concentration 1X = 120 fmol/μl), neurotensin (1672.91 Da, 1X = 80 fmol/μl) and ACTH 18–39 fragment (2465.19 Da, 1X = 100 fmol/μl). Each sample was analyzed six times.

**Figure 5:**
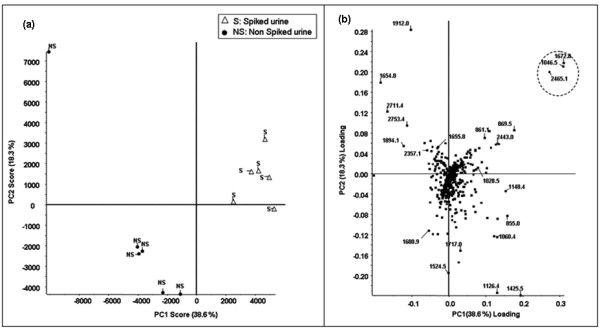
**PCA analysis of spiked and not-spiked healthy urine**. (a) Plot of the sample scores for PC1 and PC2 obtained from PCA analysis of the replicate analysis of a healthy volunteer's urine sample spiked (open triangle) or not (black circle) with calibrator peptides (Human angiotensin II (1046.54 Da 1X = 120 fmol/μl), neurotensin (1672.91 Da, 1X = 80 fmol/μl) and ACTH 18–39 fragment (2465.19 Da, 1X = 100 fmol/μl)). (b) Plot of the loadings for PC1 and PC2 for spiked and non-spiked samples (spiked peptide loadings are circled).

The analysis of 6 replicates of urine aliquots spiked or not with 3 standard peptides at increasing concentrations showed that their m/z intensity was linearly correlated with their concentration (R^2 ^> 0.96) in the range of 10 to 120 fmol/μl (Figure 4). The precision of the measure evaluated as mean CV % between the six replicates was 22.4 ± 12.5%, 10 ± 2% and 16.3 ± 7% for m/z 1046.54, 1672.94, and 2465.19 respectively. Moreover, spiked and non-spiked urine aliquots were totally discriminated by PCA, with PC scores of 38.6%, 18% and 10.3% for PC1, PC2, and PC3, respectively. Figure 5a shows the plot of sample scores for PC1 and PC2. All six replicates of spiked urine are clustered, as well as 5 out of 6 of the non-spiked urine replicates. Furthermore, the plot of the loading values for PC1 and PC2 (Figure 5b) shows that the most discriminative ions with the highest positive loadings on PC1 and PC2 are the spiked peptides. The spiked peptides were the top ranked discriminative peptides according to their PC loadings at the highest concentration (120–100 fentomole/μl) but not at their lowest (20–30 fentomole/μl), although they were still among the top ten of the most discriminative ions. The loadings of spiked peptides according to their concentration are summarized in Table 1. In addition, PCA classified correctly spiked and non-spiked urine aliquots at each spiked peptide concentration (data not shown). Good correlation was found between spiked peptide concentrations and m/z intensity, even though it is known that MALDI ionization yields poor reproducibility of peak intensity, particularly due to poor inter-spot crystallization reproducibility [14]. Quantification precision (CV%: 10–22%) was sufficient here to allow semi-quantitative estimation in the low fentomole range. The use of an internal peptide calibration using Glu^1^-Fibrinopeptide B diluted in the matrix for precision purposes and correction of matrix crystallization variability was investigated, but not pursued because of its important ion suppression effect on the other analytes, particularly at low concentrations (data not shown). However, our MS data acquisition strategy consisted in recording MS spectra resulting from 800 laser shots (accumulation of 50 MS spectra at each of 16 random locations on a spot, i.e. 16 sub-spectra), thus improving signal-to-noise ratio and sensitivity, as well as quantification precision.

**Table 1:** PCA results for urine aliquots spiked or not with pseudo-markers peptides at increasing concentrations

**Pseudo-markers**	**Concentration (fmol/μl)**	**PC1**	**PC2**	**PC3**	**m/z Rank**
**Low level**					
PC (%)		33.8	17.7	14.8	-
1672.83	20	0.31	0.22	0.04	4
1046.46	30	0.31	0.21	0.08	2
2465.08	25	0.27	0.20	0.05	9

**Medium level**					
PC (%)		31.1	26.0	15.7	-
1672.83	40	0.19	0.15	0.24	2
1046.46	60	0.13	0.13	0.30	3
2465.08	50	0.24	0.15	0.15	4

**High level**					
PC (%)		38.6	18.3	10.3	-
1672.83	80	0.14	0.19	0.24	1
1046.46	120	0.15	0.20	0.14	2
2465.08	100	0.10	0.16	0.07	3

The rank of each standard peptide in the complete dataset is given according to the sum of their loading absolute values in the 3 retained PCs

## Conclusion

This study showed the beneficial effect of a dual decoupling strategy between Nano-LC MALDI-TOF and TOF/TOF mass spectrometry on overall analytical time. Associated with PCA, this method allowed successful discrimination of urine samples spiked or not with low fentomole/μl synthetic peptides used as pseudo-biomarkers. This method is currently being applied to the discovery phase of potential urine biomarkers of graft rejection in kidney transplant patients.

## Abbreviations

PCA: Principal component analysis; SPE: Solid phase extraction; TFA: Trifluoro acetic acid; FA: Formic acid; MALDI TOF/TOF: matrix assisted laser desorption ionization time of flight/time of flight.

## Authors' contributions

KB carried out samples processing, LC-MS and data analyses and manuscript writing; PM participated in the design of the study and manuscript writing; JPR and YLM provided urine samples and participated in data analysis; LNG conceived the study and participated in manuscript writing. All authors read and approved the final manuscript.

## Supplementary Material

Additional file 1 **LC-MALDI chromatograms dataset:**. contains aligned and filtered data of LC-MALDI analysis from spiked urine with pseudo-biomarkers at 3 different concentrations (0.25×, 0.5×, 1×) and from not spiked urine (0×). The table contains detected masses and their chromatographic retention time in the first column with the format (m/z_RT) and their intensity in each hexareplicate urinary sample. Click here for file

Additional file 2 **MSMS spectra and fragmentation evidence of urinary spiked peptides: **Observed MSMS spectrum of pseudo-biomarker with m/z 1046.54. Click here for file

Additional file 3 **MSMS spectra and fragmentation evidence of urinary spiked peptides: **Mascot interpretation of MSMS fragmentation spectrum of pseudo-biomarker with m/z 1046.54. Click here for file

Additional file 4 **MSMS spectra and fragmentation evidence of urinary spiked peptides: **Observed MSMS spectrum of pseudo-biomarker with m/z 1672.91. Click here for file

Additional file 5 **MSMS spectra and fragmentation evidence of urinary spiked peptides: **Mascot interpretation of MSMS fragmentation spectrum of pseudo-biomarker with m/z 1672.91. Click here for file

Additional file 6 **MSMS spectra and fragmentation evidence of urinary spiked peptides: **Observed MSMS spectrum of pseudo-biomarker with m/z 2465.19. Click here for file

Additional file 7 **MSMS spectra and fragmentation evidence of urinary spiked peptides: **Mascot interpretation of MSMS fragmentation spectrum of pseudo-biomarker with m/z 2465.19. Click here for file
