# Supplementary figures and images for: A new strategy for faster urinary biomarkers identification by Nano-LC-MALDI-TOF/TOF mass spectrometry
Source: BMC Genomics. 2008 Nov 14;9:541. doi: 10.1186/1471-2164-9-541 (PMC2596142; doi:10.1186/1471-2164-9-541)

MSMS spectra: precursor = 1046.54 Da Angiotensin II human.  
Sequence: DRVYIHPF

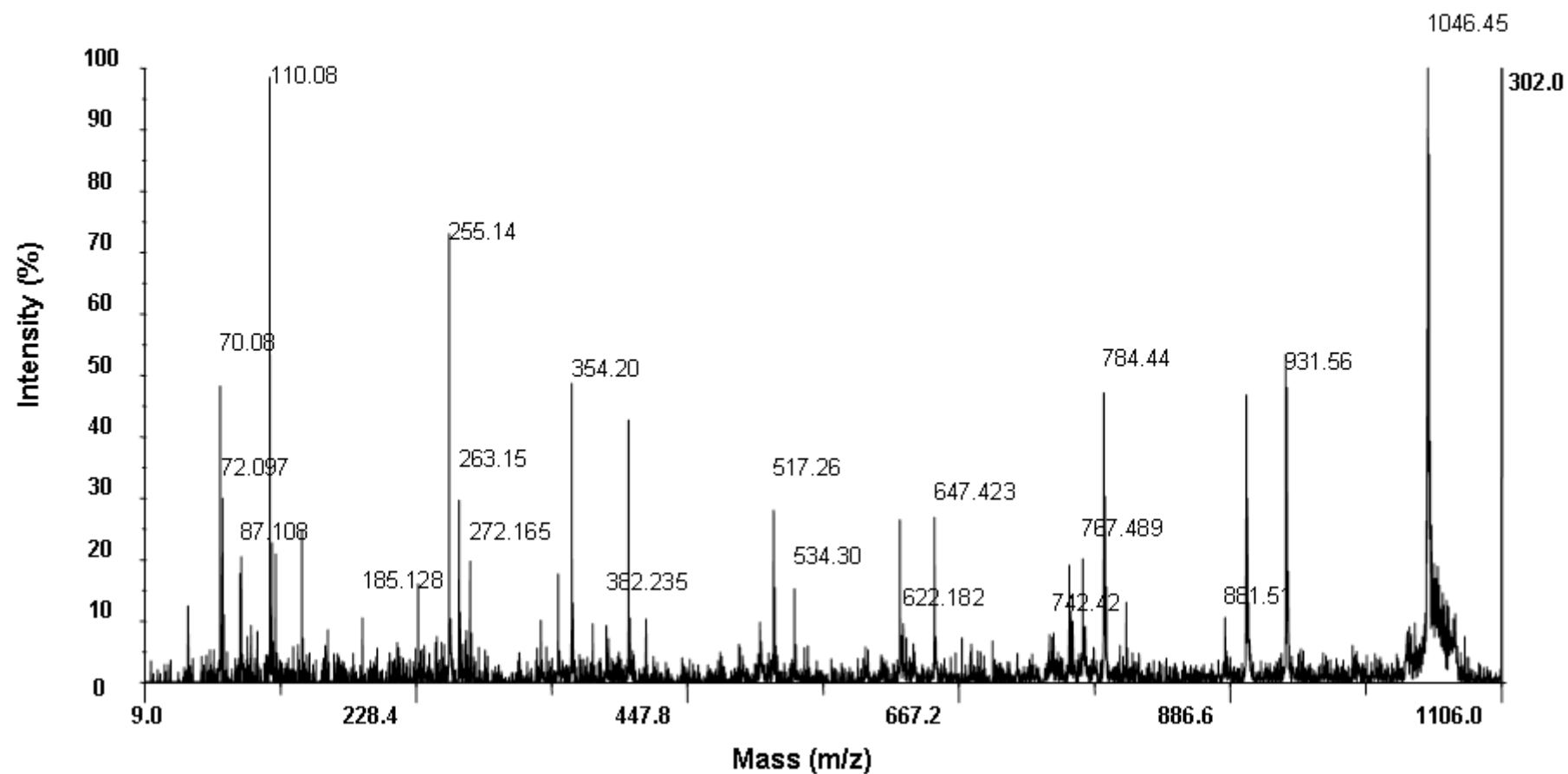

Supplement: Additional file 2 — MSMS spectra and fragmentation evidence of urinary spiked peptides: Observed MSMS spectrum of pseudo-biomarker with m/z 1046.54. [file 1471-2164-9-541-S2.pdf]

Fragmentation evidence: precursor = 1046.54 Da Angiotensin II human.  
Sequence: DRVYIHPF

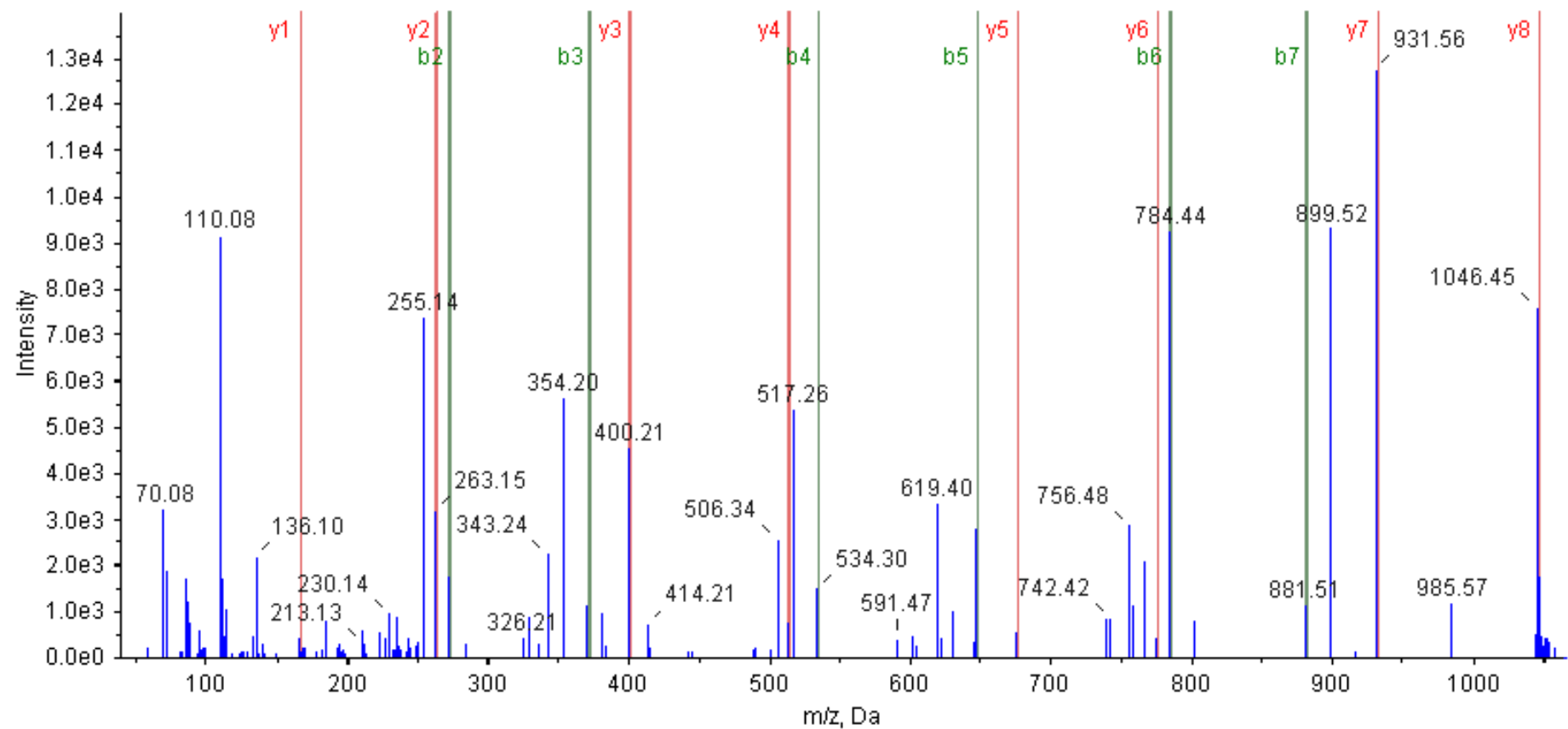

Supplement: Additional file 3 — MSMS spectra and fragmentation evidence of urinary spiked peptides: Mascot interpretation of MSMS fragmentation spectrum of pseudo-biomarker with m/z 1046.54. [file 1471-2164-9-541-S3.pdf]

MSMS spectra: precursor = 1672.91 Da Neurotensin.

Sequence: QLYENKPRRPYIL

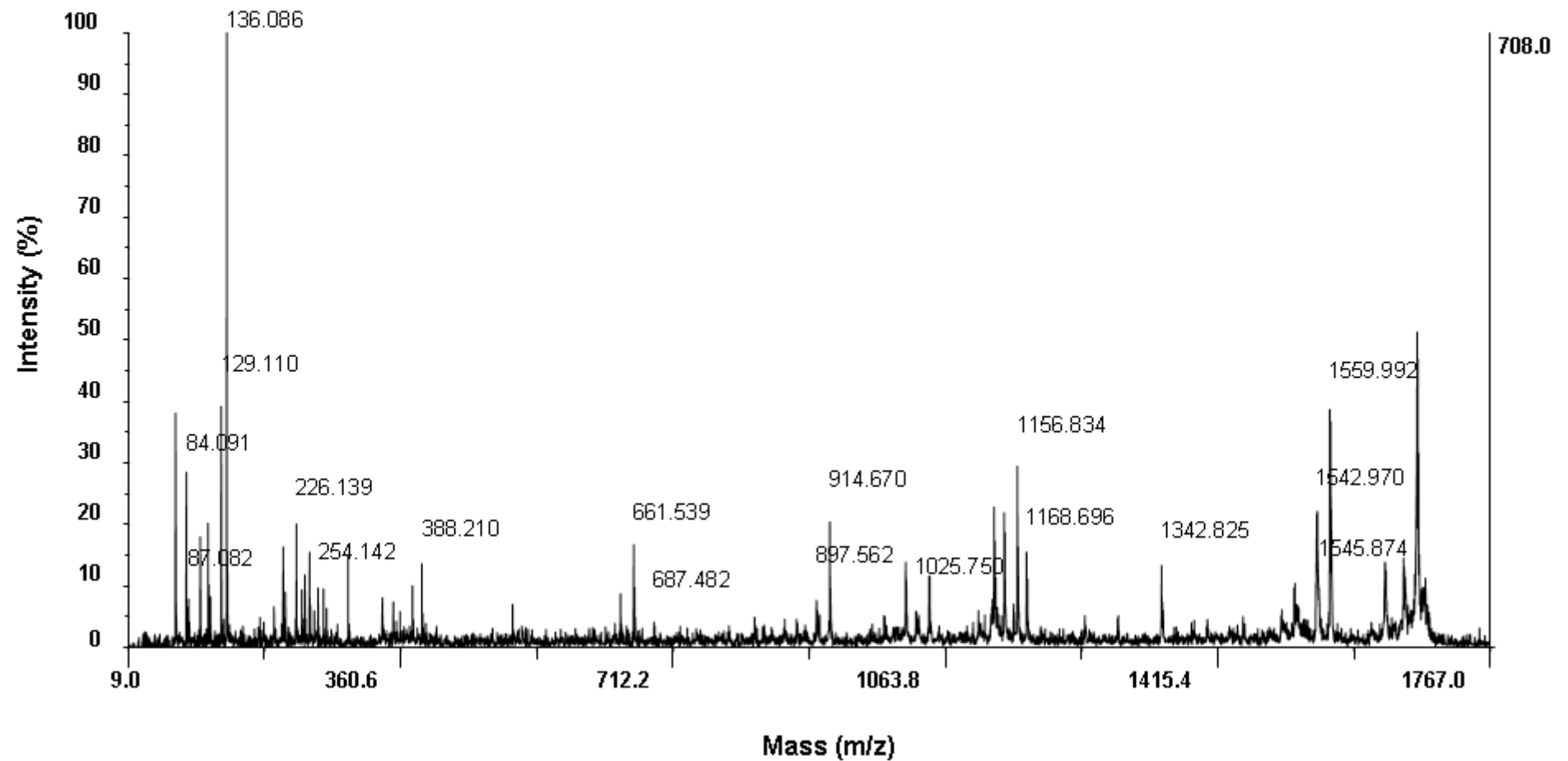

Supplement: Additional file 4 — MSMS spectra and fragmentation evidence of urinary spiked peptides: Observed MSMS spectrum of pseudo-biomarker with m/z 1672.91. [file 1471-2164-9-541-S4.pdf]

Fragmentation evidence: precursor = 1672.91 Da Neurotensin.  
Sequence: QLYENKPRRPYIL

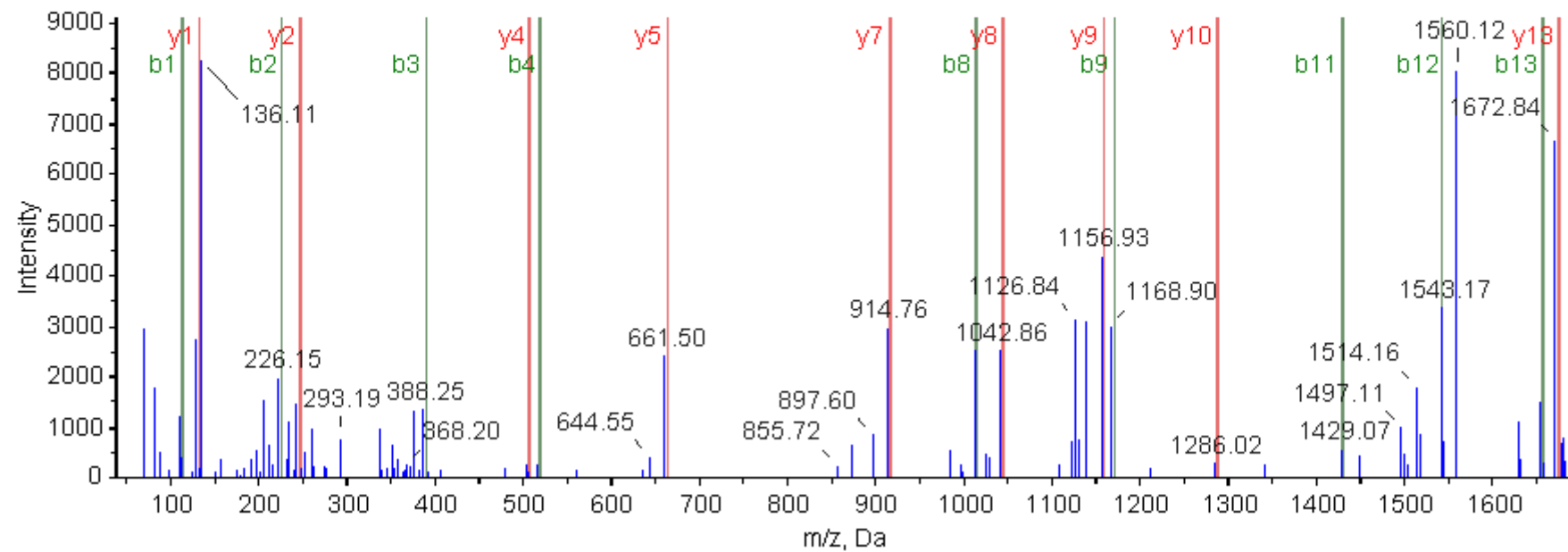

Supplement: Additional file 5 — MSMS spectra and fragmentation evidence of urinary spiked peptides: Mascot interpretation of MSMS fragmentation spectrum of pseudo-biomarker with m/z 1672.91. [file 1471-2164-9-541-S5.pdf]

MSMS spectra: precursor = 2465.19 Da ACTH clip [18-39].

Sequence: RPVKVYPNGAEDESAFAFPLEF

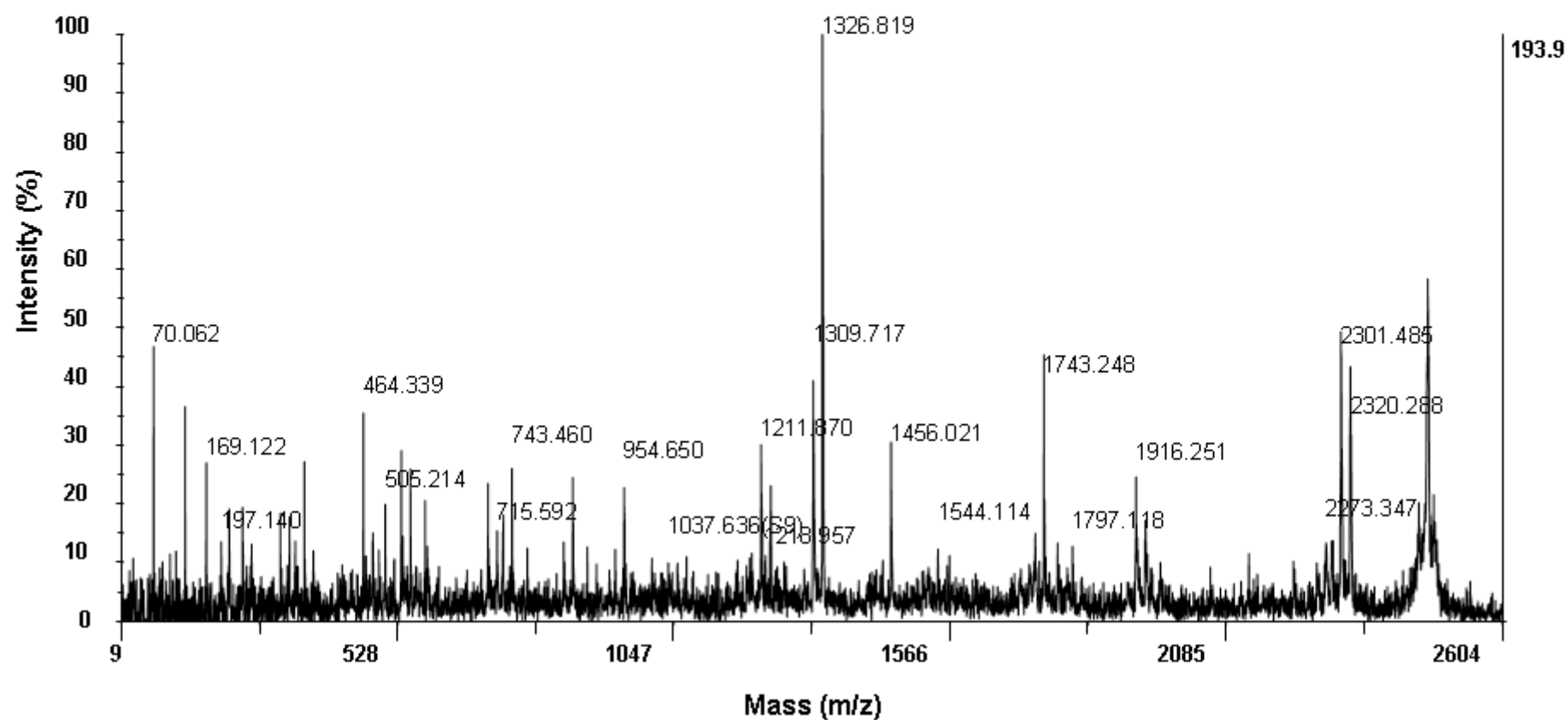

Supplement: Additional file 6 — MSMS spectra and fragmentation evidence of urinary spiked peptides: Observed MSMS spectrum of pseudo-biomarker with m/z 2465.19. [file 1471-2164-9-541-S6.pdf]

Fragmentation evidence: precursor = 2465.19 Da ACTH clip [18-39].

Sequence: RPVKVYPNGAEDESAEAFPLEF

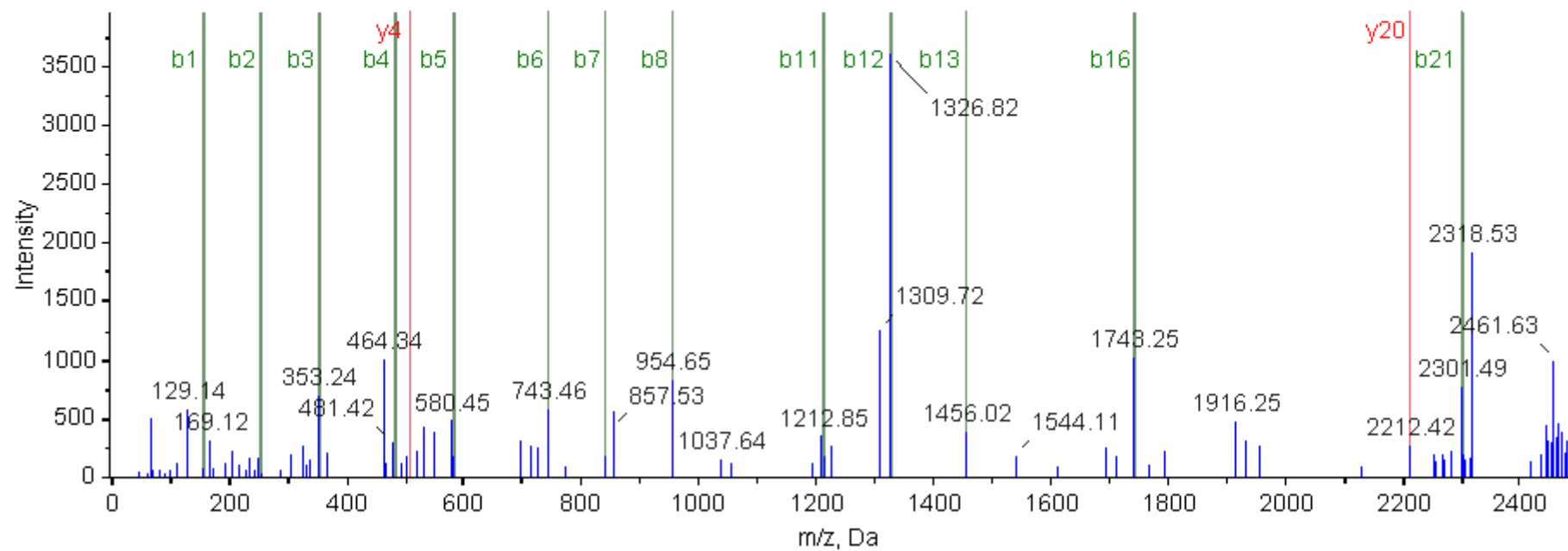

Supplement: Additional file 7 — MSMS spectra and fragmentation evidence of urinary spiked peptides: Mascot interpretation of MSMS fragmentation spectrum of pseudo-biomarker with m/z 2465.19. [file 1471-2164-9-541-S7.pdf]
